# Finite element analysis of the use of two new types of internal fixation for acetabular fractures

**DOI:** 10.1186/s13018-023-04337-9

**Published:** 2023-11-07

**Authors:** Guanggui Lv, Xianglong Chen, Hui Wu, Guilong Wu, Yinglin Huang, Guixiong Huang

**Affiliations:** 1 Department of Pelvic Surgery, Yulin Orthopedic Hospital of Integrated Traditional Chinese and Western Medicine, Yulin, Guangxi Province People’s Republic of China; 2 Department of Orthopedics, The First People’s Hospital of Fangchenggang City, Fangchenggang, Guangxi Province People’s Republic of China; 3 Department of Radiology, Pingxiang People’s Hospital, Pingxiang, Guangxi Province People’s Republic of China; 4 Department of Radiology, Guigang People’s Hospital, Guigang, Guangxi Province People’s Republic of China; 5 Department of Orthopaedics, Union Hospital, Tongji Medical College, Huazhong University of Science and Technology, Wuhan, Hubei Province People’s Republic of China

**Keywords:** Acetabular fracture, Finite element analysis, Union plate

## Abstract

**Background:**

Both-column fracture is a common type of acetabular fracture and is sometimes accompanied by a comminuted fracture of the quadrilateral area. Such fractures are difficult to anatomically reduce and securely fix. In this study, the authors compared the application value and mechanical properties of the Bespoke 3D-printed titanium alloy plates and Union Plate in acetabular both-column fractures.

**Methods:**

A both-column fracture model of the acetabulum was established, and the Bespoke 3D-printed titanium alloy plates, Union Plate and a common reconstruction plate were used for fixation. External loads were applied to the model at different angles, and the effects on the plates and the stress and displacement of the screws were determined.

**Results:**

Under different states of hip joint activity, the maximum stress experienced by the Bespoke 3D-printed titanium alloy plates and Union Plate was significantly smaller than the maximum stress experienced by the common reconstruction plate. The Bespoke 3D-printed titanium alloy plates experienced the lowest maximum stress under different hip joint motions. There was no statistically significant difference between the maximum displacement of the Bespoke 3D-printed titanium alloy plates and Union Plate and that of the common reconstructed plate.

**Conclusions:**

The design of the Bespoke 3D-printed titanium alloy plates imparts a smaller maximum stress and better mechanical properties when repairing acetabular both-column fractures.

## Background

Acetabular fractures are common in falls from heights and car accidents [1, 2]. Among them, the both-column fracture is the most common type, accounting for approximately 20% of acetabular fractures [3]. According to recent studies, the number of acetabular fractures has increased in recent years [4]. Both-column fractures are accompanied by fractures in the quadrilateral area, which can sometimes manifest as comminuted fractures. This type of fracture is common in elderly patients [4]. Traditional plates generally cannot completely fix fractures in the quadrilateral area; since fractures in this region also involve the articular surface, poor surgical reduction will lead to poor surgical results. Common complications are traumatic arthritis and avascular necrosis of the femoral head [5]. Recent studies have shown that the satisfaction rate for the treatment of acetabular fractures is between 70 and 80% [6].

Acetabular fractures are extremely unusual in elderly individuals because of associated osteoporosis. A study by Park et al. [7] found that acetabular fractures in some elderly patients involved acetabular roof (24.2%) and posterior wall (23%) impaction. Sometimes acetabular fractures are accompanied by comminuted fractures in the quadrilateral area [8], which are difficult to fix using conventional internal fixators. The most common postoperative complication from this procedure is traumatic hip arthritis [9]. Although elderly patients often have different underlying diseases and limited tolerance for surgery, better clinical results can be obtained for elderly patients with acetabular fractures and surgical indicators with surgery than with conservative treatment [10]. To this end, Lin et al. [11] designed Bespoke 3D-printed titanium alloy plates, and Guo et al. [12] designed the Acetabular Union Plate series of plates to be used to fix fracture displacement in the quadrilateral area of the acetabulum, filling the gap in the literature to a certain extent. However, the roles that these plates fill in the fixation of fractures in the quadrilateral area of the acetabulum remain unknown.

The main purpose of this study is to use finite element analysis to compare the mechanical properties of Bespoke 3D-printed titanium alloy plates and Union Plates (the term used in this study for one of the Union Plate series of plates consisting of an integrated plate above the arcuate line) when applied to a relatively simple both-column fracture of the acetabulum. Assuming that the fracture reduction method is anatomical reduction, we also sought to determine which of the internal fixation methods produced better effects.

## Methods

For this study, a healthy adult male (height 175 cm, age 26 years old) was recruited to undergo a pelvic CT scan. CT (Siemens, Germany) had 64 rows, the reconstruction slice distance was 0.625mm, and the image matrix was 512 × 512. The images were then saved in DICOM format for future use. To create the model, the data were imported into Mimics 21.0 software (Materialise, Leuven, Belgium), and the left iliac model was constructed. This study used CT Bone function that came with the Mimics software. After selecting the target iliac bone, a Mask was formed on the CT image, the redundant parts were deleted layer by layer, and the target bone was separated in the form of Mask, and then, three-dimensional reconstruction was performed using Wrap and Smooth functions modify the 3D model to be consistent with the actual bone anatomy to the greatest extent possible. Using this model, the normal iliac bone was cut to create a both-column fracture. Then, Bespoke 3D-printed titanium alloy plates, Union Plates and common reconstruction plates were produced in 3-Matic software (Materialise, Leuven, Belgium), as well as the corresponding number of screws, according to the fracture model. The above model was imported into Hypermesh 13.0 software (Altair Company, Troy, MI, USA) to separate the surface mesh and volume mesh. The data were then exported in.inp format for later use.

All fracture models were imported into Mimics 21.0 in.inp format, and the materials were then attached. The method for attaching the materials was carried out according to our previous research [12, 13] and can approximately simulate the nonisotropic characteristics of bone. This method can also divide the CT values of different bone types into 11 different materials. In this study, it is assumed that both the plates and screws have isotropic material characteristics. The element type of all models is a three-node tetrahedral element (C3D4 element). The material of the plate and screws is titanium alloy, with a Young's modulus of 110 GPa and a Poisson’s ratio of 0.316 [12, 14]. The contact properties between bone blocks are hard contact, the tangent behavior is a penalty, the coefficient between fracture blocks is set to 0.35, and the coefficient between bone and the plate or screw is 0.45 [15]. In order to reduce the calculation amount of this study, the thickness of all plates in this study was set to 2.2 mm and the width was 8mm. The diameter of the screws was 3.5 mm. But the diameter of the posterior column screw in common reconstruction plate was 6 mm.

Afterwards, the fracture model, the plates and the corresponding screws were imported into Abaqus 2021 software (Dassault; France) for calculation. The boundary conditions included fixing the pubic symphysis of the left hemipelvis and the ilium of the sacroiliac joint. The load was applied as follows:

1. The load amplitude was 2300 N [16]. Under this load, the experimental model could be destroyed.
2. To simulate a person standing, the load direction was applied to the top of the acetabulum.
3. Applied load directions were also simulated for the femoral head in abduction angles of 15°, 30°, and 45°, as well as at anteflexion angles of 15° and 30°, acting on the corresponding parts of the acetabular fossa in all models.

In this study, the Jupyter Notebook computing platform based on Python 3.8 scripts is used to perform statistical analysis. We compare whether there are significant differences in the maximum stress and/or displacement of the various devices under different external loads. Comparisons between groups were performed with the ***t*** test, and *P* < 0.05 indicated statistical significance.

## Results

**Fig. 1:**
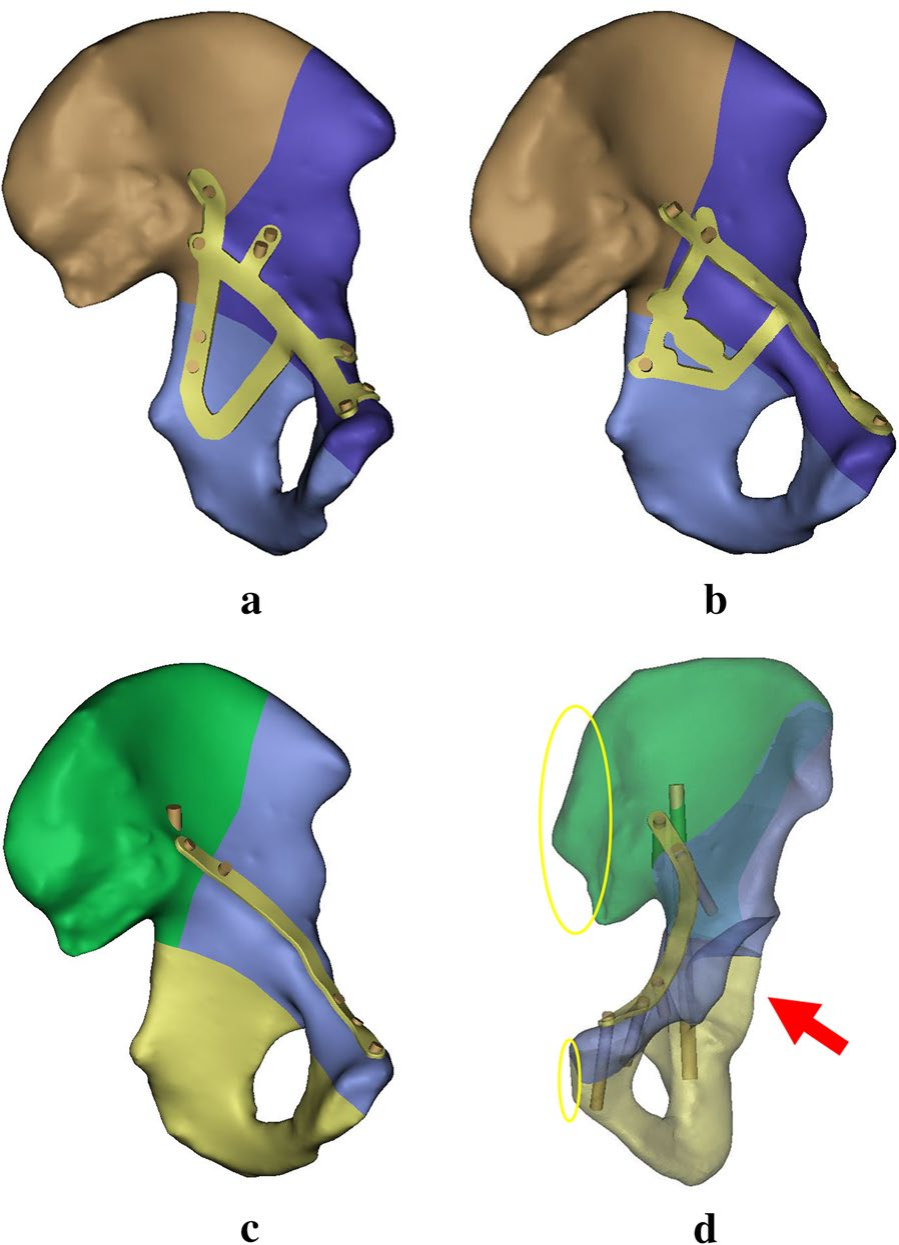
Simplified diagram of the three internal fixation devices. **a** Bespoke 3D-printed titanium alloy plates. This model is fixed by an integrated plate and 9 screws. There are wings above the arcuate line and the pubic ramus. Two screws are inserted in the posterior column of the quadrilateral area. **b** Union Plate. This model is composed of an integrated plate and 6 screws, one of which is fixed on the rear column in the quadrilateral area. **c** Common reconstruction plate. This model consists of a plate above the arcuate line and 6 screws. **d** As shown in the figure, the red arrow is the direction of the force on the model. The yellow oval area is the constrained area

In this study, the authors established a model of acetabular both-column fracture and treated it using three different fixation methods, as shown in Fig. 1. Among them, the Bespoke 3D-printed titanium alloy plates’ model has 254, 523 nodes and 1,184,169 elements, the Union Plate integrated plate model placed along the arcuate line has 351,118 nodes and 999,584 elements, and the common reconstructed plate model has 334,860 nodes and 1,108,563 elements (the numbers of these elements and nodes above are the total number of elements and nodes for each plate, screw and bone fragments).

**Fig. 2:**
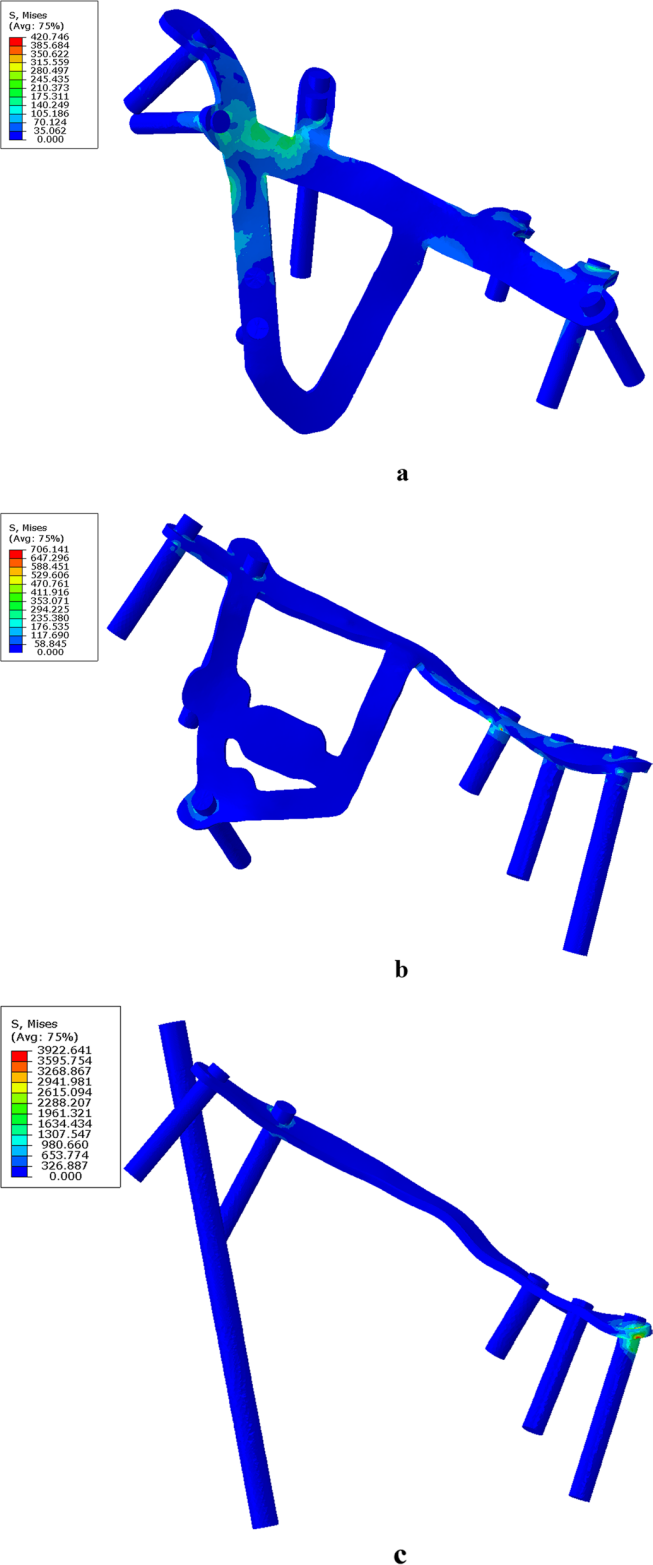
Hip joint abduction at 45° is used as an example to illustrate the stress and displacement nephogram of each model under external force. **a** Stress nephogram of the Bespoke 3D-printed titanium alloy plates’ model. The maximum stress distribution is at the junction of the posterior column and the inner arcuate line. **b** Stress nephogram of the Union Plate, with maximum stress located at the screw in the middle of the superior pubic ramus. **c** Stress nephogram of a common reconstruction plate. The maximum stress is distributed at the end of the plate near the pubic symphysis

Under different loads in different directions of hip joint movement, the maximum stress generated by the Bespoke 3D-printed titanium alloy plates’ model fluctuates between 420 and 590 Mp, and the displacement fluctuates between 0.68 and 1.29 mm. The stress in the Union Plate model fluctuates between 698 and 1025 Mp, and the displacement fluctuates between 0.31 and 1.25 mm. The stress in the common reconstructed plate model fluctuates between 3922 and 3947 Mp, and the displacement fluctuates between 0.97 and 1.09 mm. The stress nephogram of the different models are shown in Fig. 2.

**Fig. 3:**
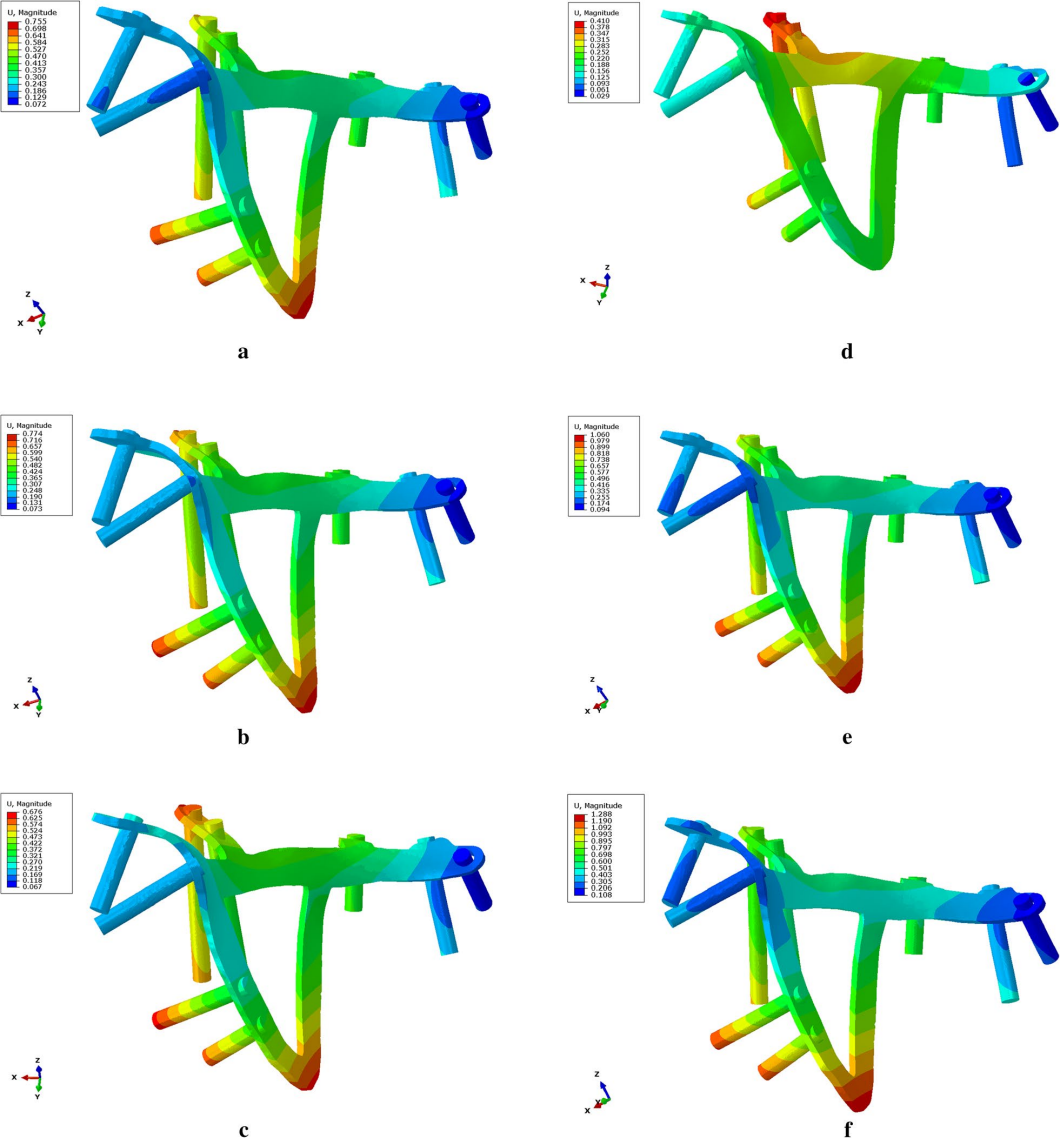
Stress nephogram of the Bespoke 3D-printed titanium alloy plates. **a**–**f** Changes in the maximum displacement of the hip joint in the upright position and at 15° abduction, 30° abduction, 45° abduction, 15° anteflexion and 30° flexion, respectively. As seen in the nephogram, as the hip joint abducts, the maximum displacement gradually shifts from the plate on the inner lower side of the blocked quadrilateral area to the screws fixed to the posterior column and the penultimate flank behind the arcuate line of the plate. As the hip joint flexes forwards, the migration of the maximum displacement is not obvious

According to the stress nephogram, the maximum stress of the Bespoke 3D-printed titanium alloy plates’ model is distributed at the inner junction of the posterior column and the arcuate line. The maximum displacement gradually moves from the plate on the inner lower side of the blocked quadrilateral area to the screws fixed to the posterior column and penultimate flank behind the arcuate line of the plate. When the hip joint moves forwards, the maximum displacement transfer is not obvious, as shown in Fig. 3. The maximum stress on the Union Plate model is located in the middle of the superior pubic ramus. As the hip joint abducts, maximum displacement occurs. The screws located at the lowest part of the medial posterior column of the quadrilateral area are gradually distributed at the pubic protuberance. As the hip joint flexes forwards, maximum stress occurs. The maximum displacement is still located at the screw at the lowest part of the posterior column on the inner side of the quadrilateral area, but no obvious migration trend is observed. The maximum stress in the common reconstruction plate model is distributed along the end of the plate near the pubic symphysis. As the hip joint abducts, the maximum displacement is located at the screw near the sacroiliac joint. When the hip joint flexes forwards, the maximum displacement tends to gradually shift to the posterior column screw. These findings are summarized in Tables 1 and 2.

**Table 1:** Stresses in different internal fixation models at different hip joint positions (MPa)

Hip position	Bespoke 3D-printed titanium alloy plates	Union plate	Common reconstruction plate
Upright position	498.62	704.12	3924.95
Abduction 15°	589.30	704.83	3934.04
Abduction 30°	592.90	701.54	3932.31
Abduction 45°	420.75	706.14	3922.64
Anteflexion 15°	541.22	698.02	3937.68
Anteflexion 30°	526.80	1024.78	3946.58
*P* value	0.05 < *P*

**Table 2:** Displacements in different internal fixation models at different hip joint positions (mm)

Hip position	Bespoke 3D-printed titanium alloy plates	Union plate	Common reconstruction plate
Upright position	0.78	0.61	1.06
Abduction 15°	0.77	0.48	1.07
Abduction 30°	0.68	0.50	1.04
Abduction 45°	0.41	0.31	0.97
Anteflexion 15°	1.06	1.01	1.08
Anteflexion 30°	1.29	1.25	1.09
*P* value	*P* > 0.05		

### Statistical analysis

According to the *t* test analysis, for all hip joint movements, the differences between the maximum stress of both the Bespoke 3D-printed titanium alloy plates and Union Plate and the maximum stress of the common reconstruction plate were significant (*P* < 0.01, *P* < 0.05, respectively). However, the maximum displacements of the Bespoke 3D-printed titanium alloy plates and Union Plate were not significantly different from the maximum displacement of the common reconstruction plate according to the*** t*** test (*P* = 0.14 > 0.05 and *P* = 0.06 > 0.05, respectively). These results for the maximum displacement are consistent with the trends observed in the biomechanical research of Fan et al. [17], indicating that the results of this study are reliable.

## Discussion

In this study, the maximum stress of the Bespoke 3D-printed titanium alloy plates when subjected to different external loads is smaller than that of the Union Plate and common reconstruction plate; statistical analysis revealed that these differences were significant. However, there was no significant difference among the three models in the maximum displacement when subjected to different external loads. It can be seen that the mechanical properties of the Bespoke 3D-printed titanium alloy plates in finite element analysis may be better than those of the Union Plate and common reconstruction plate. The maximum stress of the common reconstruction plate was relatively large and concentrated at the pubic symphysis. With the application of cyclic loads caused by walking, the internal fixation device is prone to fatigue damage [18], suggesting that screws or plates in this part may more easily loosen or break.

Since the load applied in this study was the physiological limit of the human body, the maximum stress experienced by the Bespoke 3D-printed titanium alloy plates was lower than the maximum yield stress of titanium alloy [19], but the maximum stress experienced by the common reconstruction plate exceeded its maximum yield stress. The design of the Bespoke 3D-printed titanium alloy plates may have allowed the stress to be dispersed throughout the material. Comparing the maximum stress distributions of the three models in this study, the stress experienced by the Bespoke 3D-printed titanium alloy plates was mainly located at the junction of the posterior column and the arcuate line, which is different from that of the Union Plate and common reconstruction plate, where the stress was mainly distributed at the pubic protuberance and pubic symphysis, respectively. We believe that this may be due to the side-wing design of the Bespoke 3D-printed titanium alloy plates, which has a larger contact area with the superior pubic ramus on the affected side. The area where the maximum stress is concentrated in this wing-shaped plate design is exactly in line with the frame of the rear column mentioned in the “frame-buttress theory” [12], further verifying its rationality.

Analysis of the maximum displacement in this study suggests that as the abduction angle of the hip joint increases, the location of maximum displacement migrates when external forces are not applied, moving further from the action site. However, this phenomenon is not obvious when the hip joint is flexed forwards. The reason for this phenomenon may be the combined effect of the direction of force, the position of the internal fixation screw, and the force conduction process, but further study is necessary to determine the actual underlying causes. However, during the entire process, the maximum displacement did not exceed 1.5 mm, providing a stable mechanical environment during the fracture healing process and avoiding a poor prognosis due to postoperative fracture displacement due to weak fixation [20].

The Bespoke 3D-printed titanium alloy plates and Union Plate in this study are mainly used for both-column fractures involving the acetabular region or acetabular fractures involving the quadrilateral region. Because both designs account for the fracture and displacement of the quadrilateral area caused by fracture of the acetabulum, they have a blocking effect on fractures in the quadrilateral area. This plays a further stabilizing role in the fixation of acetabular fractures and avoids the possibility of central dislocation of the femoral head after surgery due to weak fixation in the quadrilateral area. Especially for elderly patients, it can be difficult to fix comminuted fractures in the quadrilateral area [21]; thus, the two aforementioned plates are theoretically very suitable under such circumstances.

Currently, Bespoke 3D-printed titanium alloy plates are produced using selective laser melting. Lin et al. [11] conducted animal experiments and histological studies, showing that the Bespoke 3D-printed titanium alloy plates have good biocompatibility. This indicates that the plate is suitable for use in personalized treatment. Wen et al. [17] used biomechanical methods to confirm that Bespoke 3D-printed titanium alloy plates have better stiffness and yield strength than other traditional internal fixation methods, suggesting that the internal fixation from the former is relatively stable.

Chen et al. [22] conducted biomechanical analysis on a special suprapectineal QLS buttress plate (one of the Union Plate series of plates) using a model of a comminuted fracture of the quadrilateral area of the acetabulum. Different design styles of the Union Plate were used. Compared with traditional internal fixation devices, the advantages of the Union Plate series in fixing the quadrilateral area of the acetabulum were described as effective blocking and strong fixation in the region. In this study, it was found that the maximum stress of this plate was larger than that of the Bespoke 3D-printed titanium alloy plates, and the difference was statistically significant. Therefore, under the same conditions, we recommend using Bespoke 3D-printed titanium alloy plates to fix fractures in the quadrilateral area, as this may avoid loosening or breakage of the internal fixation materials postoperatively to a certain extent.

## Limitations

Since the bone models in this study are relatively simple, missing soft tissue parts such as the muscles around the joints, joint capsules, and articular cartilage, the research results may not fully reflect biological reality after fracture fixation. In this study, only the stress of the model statically was analyzed, and dynamic analysis results are lacking. Further research is needed to confirm the accuracy of the model. The inherent shortcoming of the finite element analysis method itself lies in the approximate nature of the simulation. The calculation results are affected by the type of model element, the size of the element and the performance of the computer. The calculation result is only an approximate value, not a real value, and can only reflect the changes in the stress of the model. At present, the results of this study cannot reflect the stability of internal fixation when the hip joint is subjected to cyclic loading after surgery or the conditions when fatigue damage occurs. The presented simulation assumes locking screw fixation which, from a manufacturing point of view, is not trivial to achieve with a 3D printed plate. Fractures were simulated as straight osteotomy cuts which is not representative of fractures observed clinically.

## Conclusions

In summary, according to the results of this study, the wing-like structure of the Bespoke 3D-printed titanium alloy plates helps stabilize their mechanical properties under different external loads and makes them more suitable for the internal fixation of quadrilateral fractures.

## Data Availability

The datasets used and/or analyzed during the current study available from the corresponding author on reasonable request.
